# Testing the cognitive-behavioral model of prolonged grief disorder (PGD): distinct and shared pathways to PTSD and depression

**DOI:** 10.1186/s40359-025-03078-0

**Published:** 2025-07-07

**Authors:** Emrah Keser, Paul A. Boelen

**Affiliations:** 1 Department of Psychology, TED University, Ziya Gökalp No: 48, Ankara, 06420 Turkey; 2 Department of Clinical Psychology, Utrecht University, Ultrecht, Netherlands

**Keywords:** Bereavement, Grief, Prolonged grief, Cognitive-behavioral model of prolonged grief

## Abstract

**Background:**

The cognitive-behavioral model of prolonged grief [1] posits that the interaction between autobiographical memory deficits, negative cognitions, and avoidance contributes to the onset and persistence of prolonged grief. This study tested the model’s assumptions with respect to symptoms of Prolonged Grief Disorder (PGD), Post-Traumatic Stress Disorder (PTSD), and depression in bereaved groups who experienced expected and unexpected loss, separately.

**Methods:**

The study sample comprised 728 Turkish individuals who had lost a first-degree family member. A set of self-report measures was administered to participants, including the Prolonged Grief Disorder Scale (PG-13-Revised), the Posttraumatic Stress Disorder Checklist for DSM-5 (PCL-5), the Patient Health Questionnaire-9 (PHQ-9), the Grief Cognitions Questionnaire (GCQ-SF), the Depressive and Anxious Avoidance in Prolonged Grief Questionnaire (DAAPGQ), and the Experienced Unrealness Scale (EUS).

**Results:**

Our findings indicate that while variables within the cognitive-behavioral model exhibit moderate to strong intercorrelations, they nonetheless represent statistically distinct constructs. These variables significantly explain the variance in symptoms of PTSD, depression, particularly PGD, in groups confronted with expected and unexpected loss. While negative cognitions and avoidance were significantly associated with all three outcomes, “a sense of unrealness” (representing autobiographical memory deficits) was significantly related only to PGD.

**Conclusion:**

These results support the cross-cultural applicability of the cognitive-behavioral model and suggest that it can be used to explain the development of grief-related outcomes, such as PTSD and depression, beyond PGD symptoms. Although the cognitive model variables negative grief cognition and avoidance showed significant associations with grief-related outcomes, a sense of unrealness appeared to be specific to PGD only. It is considered that taking this finding into account in clinical practice may be beneficial.

## Background

The death of a family member is a devastating life event that nearly everyone experiences at some point in their lives. Bereaved individuals may develop cognitive, emotional, behavioral, and physical symptoms in response to this devastating event [2]. For most bereaved individuals, grief symptoms gradually diminish over time, and social and occupational functioning is eventually restored [3]. However, some individuals develop mental disorders such as Prolonged Grief Disorder (PGD), Post-Traumatic Stress Disorder (PTSD), or depression [4, 5]. Various risk factors—such as the unexpected or violent nature of death, low education, low income, being female, insecure attachment, and the quality of the pre-death relationship—can influence the prevalence of PGD, which has been reported to range between 1.2% and 10% [6–10]. The prevalence rates of PTSD and depression among individuals who have experienced a loss are as high as those of PGD, and there is a strong comorbidity between PGD, PTSD, and depression [11–13]. For instance, in a study of bereaved individuals following a natural disaster, the prevalence of PTSD and depression was found to be 29.3% and 37.8%, respectively [13]. Moreover, a comprehensive review by Komischke-Konnerup et al. [12] reported that 63% of individuals with PGD also experienced co-occurring depression, and 49% had post-traumatic stress symptoms. The question of why some individuals adapt to life after loss while others develop disorders such as PGD has been explored for many years. Cognitive-behavioral models, such the cognitive-behavioral conceptualization put forth by Boelen et al. [1] and the cognitive-attachment model from Maccallum and Bryant [14] and other models [15, 16], offer explanations for how normal grief can develop into psychopathology. These models have similarities, including a shared emphasis on the roles of autobiographical memory, negative cognitions, and avoidance. This article will focus on Boelen et al.‘s [1] cognitive-behavioral model of prolonged grief.

### A cognitive-behavioral conceptualization of PGD

This model explains the transformation of ordinary grief symptoms into prolonged grief through three interrelated components. The first component involves the inability to integrate new information about the deceased’s physical absence, the permanence of separation, and the irreversibility of death into autobiographical memory, which stores information about the self, the deceased, and their relationship. A sense of unrealness is considered a manifestation of the autobiographical memory deficit [1, 17]. The second component focuses on global negative beliefs and misinterpretations [1, 18]. Global negative beliefs about the self, life, and the future (e.g., “*I am weak and worthless*”, “*My life is meaningless*”, “*The future is hopeless*”) and negative interpretations of grief symptoms (e.g., “*If I fully experience my feelings or accept the death*, *I will lose control or go crazy*”) are thought to interfere with healthy adjustment and to fuel PGD symptoms. The final component is depressive and anxious avoidance. Anxious avoidance involves staying away from situations, places, people, or objects that could confront the individual with the reality of death. This avoidance stems from the belief that facing the reality of death would be unbearable or lead to a loss of control or sanity due to the pain it causes. Depressive avoidance, on the other hand, is characterized by inactivity, withdrawal, and avoidance of social activities, often driven by the belief that such activities are no longer meaningful or enjoyable following the loss of a loved one [1, 17].

Although there are significant differences in the scope and measurement of variables proposed by various cognitive models, both longitudinal [19–21] and cross-sectional studies [17, 22, 23] have demonstrated that autobiographical memory deficits, negative cognitions, and avoidant coping behaviors are associated with PGD symptoms. Some studies have identified significant differences in the correlations between depressive and anxious avoidance strategies and PGD, depression, and PTSD [22, 24]. For example, Treml et al. [24] found that depressive avoidance was strongly associated with prolonged grief symptoms (*r* =.74) and moderately associated with depression symptoms (*r* =.42). In contrast, anxious avoidance was moderately associated with prolonged grief symptoms (*r* =.54) but only weakly associated with depression symptoms (*r* =.29). Similarly, another study [22] demonstrated that depressive avoidance was associated with both prolonged grief and depression symptoms, but not with PTSD symptoms. Anxious avoidance, on the other hand, was associated with prolonged grief symptoms, but not with depression or PTSD symptoms. Similarly, the relationship between negative appraisals and PGD symptoms has been found to be stronger (β = 0.77) than the relationship between autobiographical memory deficits and PGD (β = 0.26) [23]. Studies using Latent Class Analyses (LCA) have explored various symptom profiles in the grief process, such as high grief, high depression, high PTSD, PGD + PTSD/Depression, low symptomatology, etc., and examined the relationships between the variables of cognitive-behavioral models and membership in these classes [21, 25–28]. The research findings indicate that an increase in autobiographical memory deficits, avoidance, and negative cognitions is associated with a higher likelihood of belonging to the high grief, PGD + PTSD or depression, or low adaptation classes [21, 25–28].

Previous research has not yet examined the covariance among autobiographical memory deficits, grief cognitions, and avoidance within a single model that tests how these three variables influence PGD symptoms in the presence of one another, as proposed by the cognitive model [1]. Similarly, differences in the relationships between the components of the cognitive model and PGD and bereavement-related PTSD and depression have not been adequately explored. Considering that symptoms of bereavement-related PTSD and depression are as common as PGD, and that many bereaved individuals who seek professional help exhibit these symptoms, it is crucial to explore the relationships between the components of the cognitive model and symptoms of PGD, PTSD, and depression simultaneously. Cognitive-behavioral interventions directly or indirectly incorporate one or more components such as exposure, story rewriting, meaning reconstruction, cognitive restructuring, and addressing avoidance [29–31]. However, the two main limitations stand out. First, not all patients benefit from these treatments [24, 31]. Second, intervention programs for emotional distress following bereavement primarily focus on PGD symptoms, while PTSD and depression are less frequently addressed [31, 32]. When applying cognitive-behavioral interventions to individuals who develop PTSD and depression either comorbidly with PGD or independently after a loss, it is not yet sufficiently clear which components of the cognitive model should be targeted. Therefore, there is a need for further understanding of the relationship between the components of the cognitive model and different bereavement outcomes, including PTSD and depression and PTSD. Additionally, data on the cognitive-behavioral model of PGD primarily comes from studies conducted in Western countries. We believe that testing the propositions of this model using data collected in Turkey—where beliefs about life after death, mourning rituals, the meaning of death, religious beliefs, and social support traditions during mourning differ from those in Western countries [33, 34] will enhance our understanding of the cross-cultural applicability of the model.

In this context, we aim to test the basic propositions of the cognitive model using structural equation modeling, with three models in which PGD, depression, and PTSD symptoms are treated as outcomes. In the theoretical models, the scores for a sense of unrealness, negative grief cognitions, and avoidance were designated as independent variables and PGD, PTSD, and depression were dependent variables. Moreover, these independent variables were defined as interrelated structures that do not fully overlap. Furthermore, given that the unexpected nature of a death has been identified as a strong predictor of more pervasive grief (Buur et al., 2024), we examined the moderating role of expectedness by testing each of our models separately in two groups, including bereaved people confronted with expected or unexpected loss, respectively.

## Method

### Participants

The sample for this study consisted of 728 bereaved individuals over the age of 18. Participants were recruited using convenience sampling. The criteria for participation in the study were as follows: (a) Having lost a first-degree family member (mother, father, child, spouse, or sibling) due to death, (b) At least 6 months had passed since the loss, (c) At most 5 years had passed since the loss. The mean age of the final sample was 42.87 (SD = 12.36), and the mean time since the loss was 30.05 (SD = 17.32) months. Other descriptive information about the sample is presented in Table 1.

**Table 1:** Demographic characteristics of the sample (*N* = 728)

		Frequency	%
Gender			
	Female	609	83.7
	Male	110	15.1
Education Level			
	Primary/Middle School	21	1.9
	High School	93	12.8
	University student	70	9.6
	Graduate	392	53.8
	Postgraduate	151	20.7
Income Level			
	Low	63	8.7
	Middle	564	77.5
	High	101	13.9
Marital Status			
	No relationship	208	28.6
	Have a relationship but not married	91	12.5
	Married	429	58.9
Kinship (deceased person was a…)			
	Mother	219	30.1
	Father	314	43.1
	Child	45	6.2
	Spouse	42	5.8
	Sibling	108	14.9
Cause of death			
	Traffic accident	32	4.4
	Cancer	182	25.1
	Celebral hemorrhage	61	8.4
	Hearth attack	158	21.8
	Hypertension or sugar	65	9.0
	Suicide	16	2.2
	Homicide	1	0.1
	Natural disasters	28	3.9
	Terror attack	2	0.3
	Work accident	4	0.5
	COVID-19	60	8.3
	Organ failure	22	3.0
	Other	95	13.1
Expectedness			
	Unexpected	387	53.2
	Expected	341	46.8

*Sample sizes differed due to occasional missing values

### Meassures

#### Prolonged grief disorder (PG-13-revised) scale

PG-13-R was developed to measure the severity of PGD symptoms [35]. Accordingly, PG-13-R items assess yearning, preoccupation, identity disruption, disbelief, avoidance, intense emotional pain, difficulty with reintegration, emotional numbness, a sense that life is meaningless, and loneliness. Respondents rate the presence of symptoms during the last months on 5-point scales (1 = Not at all, 5 = Overwhelmingly). Cronbach’s alpha values were found between 0.83 and 0.93 [35]. Similarly, in the current study the alpha was 0.90.

#### Posttraumatic stress disorder checklist for DSM-5 (PCL-5)

PCL-5 [36] consists of 20 Likert-type items that evaluate the symptoms of PTSD as per DSM-5 [37]. Respondents were instructed to rate items while keeping their loss in mind as the anchor even. Items were rated on 5-point scales ranging from 0 = not at all, 4 = Extremely. PCL-5 has demonstrated strong internal consistency (*α =* 0.94) and test-retest reliability (*r =.*82) (Blevins, et al., 2015). The Turkish adaptation conducted by Boysan et al. [38] confirmed the scale’s 4-factor structure (re-experiencing, avoidance, negative alterations in cognition and mood, hyperarousal, ) and demonstrated high composite reliability for both community and clinical samples (α = 0.94 and 0.97, respectively). Cronbach’s alpha value was found to be 0.95 in the current study.

#### The patient health questionnaire-9 (PHQ-9)

The Patient Health Questionnaire-9 (PHQ-9) [39] consists of 9 Likert-type items that measure the severity of depression symptoms (0 = not at all, 3 = nearly every day) Cronbach alpha value was 0.84 in the Turkish adaptation study [40] and 0.91 in the current study.

#### Grief cognitions questionnaire– short form (GCQ-SF)

The GCQ is a 38-item Likert-type scale (0 = strongly disagree, 5 = strongly agree) consisting of nine subscales [41]. In the current study, GCQ-SF, a 19-item short form of the GCQ comprising the self, life, future, and threatening interpretations subscales, was used. The GCQ includes items related to self (e.g., *“Since [–] is dead*,* I think I am worthless*,*”* or *“Since [–] is dead*,* I am of no importance to anybody anymore”*), future (e.g., *“My wishes for the future will never be fulfilled*,” or *“In the future*,* I will never be truly happy again”*), life (e.g., *“Life has got nothing to offer me anymore*,” or *“My life is useless since [–] died”*), and items concerning the evaluation of grief symptoms as a threat (e.g., *“If I allow my feelings to surface*,* I will lose control*,*”* or *“If I let go of my emotions*,* I will go crazy”*). The Cronbach alpha of the GCQ-SF was calculated as 0.96 in the current study.

#### The depressive and anxious avoidance in prolonged grief questionnaire (DAAPGQ)

The DAAPGQ [22] consists of 9 Likert-type items developed to measure depressive (e.g., *I develop very few new activities since [–] died*,* because I am unable to do so*) and anxious avoidance (e.g., *I avoid to dwell on painful thoughts and memories connected to his/her death*). Respondents rate their agreement with items on 8-point Likert scales with anchors 1 = Not at all true, 8 = Completely true. The Turkish form of the scale was adapted by Cesur (2017). Cronbach’s alpha values ​​of the depressive and anxious avoidance subscales were found to be 0.95 and 0.87 in Boelen and Van den Bout’s [22] study and 0.93 and 0.85 in the current study.

#### Experienced unrealness scale (EUS)

The EUS measures the level of integration of reality and irreversibility of anxiety into autobiographical memory. The scale consists of 5 Likert-type items (e.g., *It feels unreal that he/she is gone forever*, 1 = not at all true for me, 8 = completely true for me). Cronbach’s alpha values were 0.89 in the initial validation study [17] and 0.94 in the current study.

### Procedure

Ethical approval was received from TED University Human Research Ethics Committee. The data were collected online via Qualtrics between May 2024 and October 2024. Announcements of the study were published through social media platforms and sent via email to universities in different cities in Turkey. Participation in the study was entirely voluntary, and all participants signed an informed consent form. To ensure data security, participants’ survey completion time and whether there were multiple entries from the same IP address were examined.

### Data analysis

Statistical Package for the Social Sciences (SPSS) 24 was used to conduct descriptive analyses, and AMOS version 21 was used to test structural equation modeling (SEM). The following indicators were considered to assess the fit of the three models with symptoms of PGD, PTSD, and depression included as dependent variables, respectively: Chi-Square/Degrees of Freedom (CMIN < 5), Adjusted Goodness of Fit Index (AGFI > 0.90) Comparative Fit Index (CFI > 0.90), Normed Fit Index (NFI > 0.90), and Root Mean Square Error of Approximation (RMSEA < 0.08) [42, 43].

The latent variable for the cognitive model, a sense of unrealness, was created by using the 5 items in the scale as the observed variables. The observed variables for the latent avoidance variable were the scores from the anxious avoidance and depressive avoidance subscales. The latent variable for grief cognitions was created by using the scores from the self, life, future, and threatening interpretations subscales as the observed variables. Finally, since the outcome variables did not have a sufficient number of subscales, the observed variables were created using the item parceling method [44].

Each item in the study was examined for missing values. It was observed that the missing values did not exceed 1% for any item and did not form any patterns. Missing values were filled using multiple imputations. Z-scores were calculated based on the total scores of the variables to examine outliers. It was observed that all z-scores fell within the − 4 and + 4 limits, and no cases were removed as outliers [45].

Theoretical models developed for each of the three different outcomes were tested in both the expected loss and unexpected loss groups using Multiple Group Analysis conducted with the AMOS program. Critical *z*-values for the differences in path coefficients were examined to determine whether the relationships in the expected and unexpected loss groups differed significantly. A critical *z*-value falling outside the range of -1.96 to + 1.96 was used as an indicator that the path in question differed significantly between the two groups.

## Results

### Descriptive statistics

Mean, standard deviations, and standard error values ​​for the variables of the study are presented in Table 2. Pearson correlation coefficients between the variables are presented in Table 3.

**Table 2:** Descriptive statistics

	Mean	Standard Deviation	Standard Error	Min.	Max.
PG-13-R	30.01	9.17	0.34	10	50
PCL-5	29.00	17.12	0.63	0	77
PHQ-9	19.19	6.40	0.23	9	36
GCQ Self	12.24	7.34	0.27	6	35
GCQ Life	9.72	5.66	0.21	4	24
GCQ Future	14.49	6.85	0.25	5	30
GCQ Threatening interpretation	9.78	5.56	0.20	4	22
EUS	21.96	11.01	0.40	5	40
DAAPGQ Depressive avoidance	16.37	10.11	0.37	5	40
DAAPGQ Anxious avoidance	14.97	8.12	0.30	4	32

Note. DAAPGQ: Depressive and Anxious Avoidance in Prolonged Grief Questionnaire, EUS: Experienced Unrealness Scale, GCQ: Grief Cognitions Questionnaire, PHQ-9: The Patient Health Questionnaire-9, PCL-5: Posttraumatic Stress Disorder Checklist for DSM-5, PG-13-R: Prolonged Grief Disorder Revised

**Table 3:** Pearson correlation coefficients

	1	2	3	4	5	6	7	8	9	10
1- PG-13-R		0.71*	0.66*	0.66*	0.72*	0.68*	0.67*	0.63*	0.68*	0.60*
2-PCL-5			0.72*	0.62*	0.65*	0.64*	0.61*	0.48*	0.58*	0.55*
3-PHQ-9				0.61*	0.63*	0.63*	0.53*	0.36*	0.58*	0.46*
4-GCQ Self					0.85*	0.76*	0.73*	0.42*	0.60*	0.52*
5-GCQ Life						0.86*	0.75*	0.46*	0.66*	0.55*
6-GCQ Future							0.69*	0.43*	0.61*	0.49*
7-GCQ Threatening interpretation								0.49*	0.55*	0.59*
8-EUS									0.39*	0.47*
9-DAAPGQ Depressive avoidance										0.64*
10-DAAPGQ Anxious avoidance										

Note. DAAPGQ: Depressive and Anxious Avoidance in Prolonged Grief Questionnaire, EUS: Experienced Unrealness Scale, GCQ: Grief Cognitions Questionnaire, PHQ-9: The Patient Health Questionnaire-9, PCL-5: Posttraumatic Stress Disorder Checklist for DSM-5, PG-13-R: Prolonged Grief Disorder Revised Questionnaire. *all *p* values were lower than 0.001

When the expected and unexpected loss groups were compared in terms of demographic variables, no significant differences were found in education level (χ² (9) = 14.88, *p* =.09), income level (χ² (2) = 0.33, *p* =.84), or time since loss (t (726) = 0.02, *p* =.99). However, the mean age of the bereaved was significantly higher in the expected loss group (*t* (726) = -4.66, *p* =.000). Additionally, the groups differed significantly in terms of kinship to the deceased (χ² (4) = 34.03, *p* =.000), gender of the bereaved (χ²(1) = 8.15, *p* =.004) and marital status of the bereaved (χ²(2) = 7.00, *p* =.03).

### The results of the proposed model

**Fig. 1:**
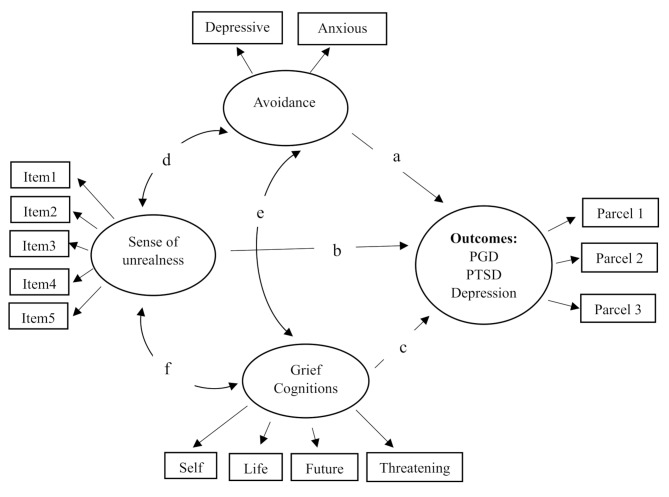
Schematic depiction of models tested in the analyses

The theoretical model tested with different outcomes is presented in Fig. 1 below.

Table 4 Presents the results obtained by testing the proposed structural model in the expected and unexpected loss groups for three different outcomes

**Table 4:** Results of the structural equation models

O	Groups	a	b	c	d	e	f	*R* ^2^	X^2^/df	GFI	IFI	CFI	RMSEA
PG13-R scores	Unexp.	0.50*	0.26*	0.28*	0.50*	0.76*	0.51*	0.81	3.22	0.92	0.97	0.97	0.05
	Exp.	0.36*	0.31*	0.37*	0.50*	0.83*	0.45*	0.81	3.22	0.91	0.96	0.96	0.05
PCL-5 scores	Unexp.	0.39*	0.12	0.30*	0.51*	0.76*	0.51*	0.53	3.12	0.92	0.96	0.96	0.05
	Exp.	0.29*	0.09	0.49*	0.53*	0.84*	0.45*	0.64	3.12	0.92	0.96	0.96	0.05
PHQ-9 scores	Unexp.	0.33*	0.02	0.43*	0.51*	0.76*	0.51*	0.50	2.87	0.92	0.97	0.97	0.05
	Exp.	0.51*	0.01	0.25*	0.50*	0.83*	0.44*	0.52	2.87	0.92	0.97	0.97	0.05

Note. * *p* <.001, O: Outcome, Unexp.: Unexpected loss group, Exp.: Expected loss group, a:Standardized regression coefficient of path a, b: Standardized regression coefficient of path b, c: Standardized regression coefficient of path c, d: Covariance coefficient of path d, e: Covariance coefficient of path e, f: Covariance coefficient of path f, X^2^/df: Chi squarre/degrees of freedom, AGFI: Adjusted goodness of fit index, CFI: Comparative Fit Index, NFI: Normal Fit Index, RMSEA: Root Mean Square Residual

As shown in Table 4, the proposed models for PGD, PTSD, and depression outcomes (Fig. 1) fit in both the expected and unexpected loss participant groups. The model with the highest explained variance by the variables of a sense of unrealness, negative cognitions, and avoidance was the one where PGD symptom scores were the dependent variable (*R*^2^ = 0.81). In the models where PTSD and depression scores were assigned as outcomes, the levels of explained variance were similar. Although each of the six tested models had excellent model fit values, the path for a sense of unrealness (b) was found to be significant only in the models where PGD scores were assigned as the outcome.

The regression coefficients were compared to determine whether the a, b, and c paths differed significantly between the unexpected and expected loss groups for the same outcome. The values found ranged from − 1.96 to + 1.96. In other words, the weights of the a, b, and c paths did not differ significantly between the expected and unexpected loss groups for the same outcome.

## Discussion

This study aimed to test the basic assumptions of Boelen et al.‘s [1] cognitive-behavioral model of prolonged grief. Consistent with the assumptions of the model, the findings showed that the following variables: (a) a sense of unrealness, representing autobiographical memory deficits, (b) negative global beliefs and misinterpretations, and (c) anxious and depressive avoidance, were strongly related to each other and to PGD symptoms, both in bereaved people confronted with expected and unexpected loss. Additionally, the variables in the cognitive-behavioral model were found to have explanatory power for the variance in bereavement-related PTSD and depression symptoms in both groups. When examining the variance explained in the expected and unexpected loss groups, it was observed that the variance explained in PGD symptoms (*R*² = 0.81, both following unexpected and expected loss) was greater than that explained in PTSD (*R*² = 0.53 and 0.64, following unexpected and expected deaths, respectively) and depression (*R*² = 0.50 and 0.52, following unexpected and expected deaths, respectively) symptoms. Both longitudinal and cross-sectional studies have shown that avoidance, negative cognitions, and difficulties in autobiographical memory integration are associated with symptoms of prolonged grief [17, 19–21, 46]. However, these studies have typically examined the role of these variables in predicting outcomes, in separate analyses. The current study expanded this prior work by exploring how autobiographical memory deficits, negative cognitions, and avoidance, when considered together, relate to each other and to grief outcomes. Additionally, nearly all studies based on the assumptions of the cognitive model have been conducted in the United States and Europe. By testing the assumptions of the cognitive model with data collected in Turkey—an environment distinct from America and Europe in terms of religious beliefs, funeral and mourning rituals, afterlife beliefs, and social support traditions [33, 34] —this study provided valuable data on the cross-cultural applicability e of the model.

Another important finding of the study is that negative cognitions and avoidance showed significant relationships with PGD, PTSD, and depression, while the sense of unrealness variable showed a significant relationship only with the PGD outcome. This finding is consistent with the findings of Boelen [17], who, after controlling for demographic variables, negative cognitions, and avoidance, found that the sense of unrealness variable was associated with PGD symptoms but not with depression. On the other hand, negative grief cognitions showed stronger relationships with both prolonged grief symptoms and depression symptoms [17]. Depressive and anxious avoidance have been shown to be associated with PGD, PTSD, and depression symptoms in numerous studies [17, 19, 22, 24, 26, 41]. Taken together, it seems that, while avoidance and cognitions—components of the cognitive-behavioral model—show similar relationships with PGD, PTSD, and depression symptoms, the sense of unrealness component exhibits a structure specific to PGD. The sense of unrealness may reflect an implicit process involving difficulties in integrating the explicit understanding of the loss’s permanence with the implicit representations of the lost person. This process appears to specifically contribute to persistent separation distress—characteristic of elevated symptoms of PGD—but not to sustained anxiety and heightened threat perception, as seen in PTSD, nor to dysphoria and anhedonia, which are commonly associated with elevated depressive symptoms [17, 47].

In our study, the correlations between the variables of the cognitive-behavioral model ranged from 0.50 to 0.84. While these values indicate a high overlap between the variables, they also provide evidence that these variables are distinct constructs. In a study by Cesur-Soysal & Durak-Batıgün [23], where all the variables of the cognitive-behavioral model were included together in a structural model, a correlation of 0.95 was found between the avoidance variable and PGD symptoms, leading them to include the avoidance variable within the PGD construct. In contrast, the current study indicated that each of the three separate variables of the cognitive model made a unique contribution to the explained variance in dependent variables that were considered.

Contrary to the findings of Cesur-Soysal and Durak-Batıgün [23], the current study found that avoidance, along with negative cognitions and autobiographical memory, made a distinct and significant contribution to prolonged grief symptoms. While both studies examined similar constructs within the cognitive-behavioral model, notable differences in sample characteristics may help explain the contrasting results. Our study employed a larger sample (*N* = 728 vs. *N* = 475) and included participants who were generally older (mean age = 42 vs. 29) and more likely to be married (58% vs. 28%). In contrast, their sample was predominantly composed of younger and mostly single individuals. More critically, the two studies differ in terms of kinship to the deceased and time since loss. All participants in our study had lost a first-degree relative, whereas in their study, the majority had experienced the loss of a second-degree relative. Furthermore, the average time since loss was considerably shorter in our sample (30 months vs. 56 months). These differences likely influenced how avoidance manifested and was measured. It is reasonable to assume that avoidance behaviors may be more prominent and detectable when the loss is more recent and involves a closer relationship, which could explain why avoidance emerged as a distinct predictor in our model.

Since the study was conducted with a cross-sectional design, our research findings cannot establish causal relationships between the variables. Additionally, the use of retrospective self-report measures may have influenced the measurements of the constructs in the study due to memory biases and the emotional state of participants at the time of assessment. Although a large sample was obtained, the generalizability of the findings is limited due to the use of a convenience sampling method. Moreover, only a few participants lost a partner or child, which is generally considered to be more distressing than loss of other close people, so generalization to people exposed to deaths of partner and children should be done with caution. As in many studies focusing on similar variables [e.g., 21, 23, 25–27], the majority of participants in the current study were female. Therefore, caution is warranted when generalizing the findings to the broader population. Future research would benefit from testing the assumptions of the cognitive model separately in male and female groups to determine whether these assumptions function similarly across genders. Although this study emphasizes that the beta values of the paths in the models tested for the expected and unexpected loss groups are similar, it is important to keep in mind that these two groups are not equivalent in terms of kinship to the deceased, gender, marital status, and age. Finally, it was observed that the relationship between the cognitive-behavioral model variables and outcomes did not differ significantly between the expected and unexpected loss groups. In this study, a distinction could not be made between objectively traumatic and non-traumatic losses because it was not possible to create two balanced groups in terms of sample size. Therefore, it is important to keep in mind that perceived expectedness was measured in this study, and the results may differ when comparing those who experienced objectively traumatic losses with those who experienced non-traumatic losses.

### Clinical implications

Since this study was conducted using a cross-sectional design, it is difficult to provide clinical implications that directly imply causal relationships. Nevertheless, we believe that some implications can be cautiously speculated upon.

The findings from this study suggest that Boelen et al.‘s [1] cognitive-behavioral model of prolonged grief may be applicable not only to PGD symptoms but also to bereavement-related PTSD and depression symptoms. Therefore, clinicians might consider referring to the cognitive-behavioral model [1] when conceptualizing cases and planning interventions, regardless of whether patients predominantly exhibit PGD, PTSD, or depression symptoms. Furthermore, the observed associations indicate that interventions aimed at facilitating acceptance of the physical absence of the deceased and the irreversibility of the loss could potentially be helpful for patients with prominent PGD symptoms. Likewise, the associations found between negative global beliefs and depression or PTSD symptoms may point to the possible value of addressing and restructuring such beliefs in clinical practice. Finally, the relevance of both depressive avoidance (e.g., withdrawal from social activities, hobbies, and relationships) and anxious avoidance (e.g., avoiding people, situations, and places that remind one of the loss) suggests that exploring these patterns in therapy might be beneficial. However, it is important to emphasize that interventions should always be tailored to the individual characteristics of the patient, as those presenting with PGD, depression, or PTSD symptoms may differ significantly in personal histories, personality traits, and cognitive styles [28, 48, 49].

## Data Availability

The datasets created and/or analyzed during this study are accessible in the ‘Figshare’ repository (https://figshare.com/) under DOI: 10.6084/m9.figshare.28784627.

## Abbreviations

AGFI: Adjusted goodness of fit index
AMOS: Analysis of moment structures
CFI: Comparative fit index
DAAPGQ: Depressive and anxious avoidance in prolonged grief questionnaire
EUS: Experienced unrealness scale
GCQ: Grief cognitions questionnaire
NFI: Normal fit index
PHQ-9: The patient health questionnaire-9
PCL-5: Posttraumatic stress disorder checklist for DSM-5
PG-13-R: Prolonged grief disorder revised
PGD: Prolonged grief disorder
PTSD: Post-traumatic stress disorder
RMSEA: Root mean square residual
